# Bactericidal and In-Vitro Cytotoxic Efficacy of Silver Nanoparticles (Ag-NPs) Fabricated by Endophytic Actinomycetes and Their Use as Coating for the Textile Fabrics

**DOI:** 10.3390/nano10102082

**Published:** 2020-10-21

**Authors:** Salem S. Salem, Ehab F. EL-Belely, Gniewko Niedbała, Maryam M. Alnoman, Saad El-Din Hassan, Ahmed Mohamed Eid, Tharwat I. Shaheen, Amr Elkelish, Amr Fouda

**Affiliations:** 1 Department of Botany and Microbiology, Faculty of Science, Al-Azhar University, Nasr City, Cairo 11884, Egypt; salemsalahsalem@azhar.edu.eg (S.S.S.); elbelely@azhar.edu.eg (E.F.E.-B.); Saad.el-din.hassan@umontreal.ca (S.E.-D.H.); aeidmicrobiology@azhar.edu.eg (A.M.E.); 2 Department of Biosystems Engineering, Faculty of Environmental Engineering and Mechanical Engineering, Poznań University of Life Sciences, Wojska Polskiego 50, 60-627 Poznań, Poland; gniewko@up.poznan.pl; 3 Biology Department, Faculty of Science, Taibah University, Al-Sharm, Yanbu El-Bahr 46429, Saudi Arabia; mnaaman@taibahu.edu.sa; 4 National Research Centre, El-Behouth St., Dokki, Giza 12622, Egypt; ti.shahin@nrc.sci.eg; 5 Botany Department, Faculty of Science, Suez Canal University, Ismailia 41522, Egypt; amr.elkelish@science.suez.edu.eg

**Keywords:** endophytic actinomycetes, Ag-NPs, green synthesis, streptomyces spp., cotton fabrics, antibacterial and cytotoxicity activities

## Abstract

An endophytic strain of *Streptomyces antimycoticus* L-1 was isolated from healthy medicinal plant leaves of *Mentha longifolia* L. and used for the green synthesis of silver nanoparticles (Ag-NPs), through the use of secreted enzymes and proteins. UV–vis spectroscopy, Fourier-transform infrared (FT-IR), transmission electron microscopy (TEM), X-ray diffraction (XRD), and dynamic light scattering (DLS) analyses of the Ag-NPs were carried out. The XRD, TEM, and FT-IR analysis results demonstrated the successful biosynthesis of crystalline, spherical Ag-NPs with a particle size of 13–40 nm. Further, the stability of the Ag-NPs was assessed by detecting the surface Plasmon resonance (SPR) at 415 nm for one month or by measuring the NPs surface charge (−19.2 mV) by zeta potential analysis (ζ). The green-synthesized Ag-NPs exhibited broad-spectrum antibacterial activity at different concentrations (6.25–100 ppm) against the pathogens *Staphylococcus aureus, Bacillus subtilis Pseudomonas aeruginosa, Escherichia coli,* and *Salmonella typhimurium* with a clear inhibition zone ranging from (9.5 ± 0.4) nm to (21.7 ± 1.0) mm. Furthermore, the green-synthesized Ag-NPs displayed high efficacy against the Caco-2 cancerous cell line (the half maximal inhibitory concentration (IC_50_) = 5.7 ± 0.2 ppm). With respect to antibacterial and in-vitro cytotoxicity analyses, the Ag-NPs concentration of 100 ppm was selected as a safe dose for loading onto cotton fabrics. The scanning electron microscopy connected with energy-dispersive X-ray spectroscopy (SEM-EDX) for the nano-finished fabrics showed the distribution of Ag-NPs as 2% of the total fabric elements. Moreover, the nano-finished fabrics exhibited more activity against pathogenic Gram-positive and Gram-negative bacteria, even after 10 washing cycles, indicating the stability of the treated fabrics.

## 1. Introduction

Nanotechnology is a promising field with the potential for integration into various industrial and biotechnological sectors, such as pharmaceuticals, agriculture, cosmetics, biodegradation, and wastewater treatment [1,2,3,4]. Nanoparticles (NPs) are referred to as those particles with a size ranging between 1 and 100 nm and with distinctive physico-chemical properties [5,6,7]. There are many different approaches to the fabrication of NPs, including physical, chemical, and biological methods [8,9,10]. Recently, due to the spread of infections in hospitals amongst both patients and medical staff, it has become urgent to develop medical textiles based on nanotechnology in order to provide smart properties such as resistance to pathogenic microbes and UV protections [11,12].

Silver nanoparticles (Ag-NPs) can be synthesized by chemical, physical, and biological methods, where the latter remains the most widely accepted and popular, due to its fast, simple, cost-effective manner, which avoids the use of hazardous chemicals and is free of hazardous by-products [13,14]. Biological synthesis of Ag-NPs can be accomplished by plants, bacteria, fungi, yeast, and actinomycetes [9,15]. Ag-NPs can be incorporated into several industrial sectors, such as medicine, pharmaceuticals, biomolecular detections, food production, agriculture, and the textile industry, due to their chemical stability, biocompatibility, catalytic activity, high conductivity, and inherent medicinal properties [1]. Furthermore, Ag-NPs are preferred over the other metal or metal oxide NPs in the textile industry, due to their high stability under UV illumination and high temperatures [16]. During textile manufacturing, Ag-NPs can be added as additives to the spinning processes used for fiber manufacture or can be utilized as a final agent for the finished textile [17,18].

Endophytes are the organisms including bacteria, fungi, and actinomycetes that can colonize internal plant tissues without invoking any adverse symptoms [19]. Endophytic microbes, especially actinomycetes, are characterized by a huge number of their metabolites, which can be utilized as reducing, capping, and stabilizing agents in the fabrication of NPs [20]. Different species of actinomycetes are used as biocatalyst for fabrication of Ag-NPs such as *Streptacidiphilus durhamensis* HGG16n [21], *Streptacidiphilus* sp. CGG11n [22], *Streptomyces noursei* H1-1 [9], *Streptomyces* spp. [23]. Ag-NPs have distinctive antimicrobial qualities which make them efficient at different particle sizes and concentrations versus a wide spectrum of Gram-negative and Gram-positive pathogens, including multidrug-resistant and biofilm-forming bacteria [24,25]. Moreover, biogenic silver nanoparticles have been extensively used as anticancer agents for the clinical management of cancer, as mediated through a cytotoxic effect due to induced oxidative stress, reducing the viability and modifying the morphology of cancer cells. Recently, many reports have focused on the potent anticancer effects of green silver nanoparticles against colon, brain, kidney, intestinal, hepatic, epidermoid, gastronomic, laryngeal, lung, and cervical cancer cell lines [26].

Therefore, in this study, we explore the efficacy of endophytic *Streptomyces antimycoticus* isolated from healthy leaves of medicinal plant *Mentha longifolia* L. as a bioreactor for the biological synthesis of Ag-NPs. The characteristics of the biosynthesized Ag-NPs are evaluated, as well as their biological activities, including antibacterial activity and cytotoxicity. Finally, a safe dose of Ag-NP coating dose, which may provide new features for fabrics, is defined.

## 2. Materials and Methods

### 2.1. Sample Collection

Healthy leaves of *Mentha longifolia* L. plants (family: Labiatae) were collected from the Rihibat Nada site (28.538664 N and 33.921423E), Saint Katherine, South Sinai, Egypt. Plant samples were placed in sterile polythene bags, transported to the laboratory in an icebox, and processed within 24 h. Plant identification was achieved in the herbarium of Botany and Microbiology Department, Al-Azhar University, Cairo, Egypt.

### 2.2. Isolation of Endophytic Actinomycetes

Surface sterilization of plant leaves was carried out by applying the five-step method described by Fouda et al. [27]. Sterilized leaves were cut into pieces (1 cm × 1 cm), plated onto starch casein agar (SCA) [28], supplemented with nalidixic acid (50 µg mL^−1^) and nystatin (25 mg mL^−1^), and incubated at 30 °C for 4 weeks. Colonies that arose from inside plant pieces and exhibited actinomycete morphologies were purified and preserved in SCA slants for further analyses. 

### 2.3. Molecular Identification of Actinomycetes Isolates

The purified cells of isolated endophytic actinobacteria were washed in saline solution (NaCl, 0.085%). Genomic DNA was extracted using the GeneJET Purification Kit (Thermo K0721, Thermo Fisher Scientific Inc., Ottawa, ON, Canada). Then, 16S-rRNA genes were amplified using Maxima Hot Start PCR Master Mix (Thermo K1051, Thermo Fisher Scientific Inc., Ottawa, ON, Canada) in 50 µL reactions using the universal primers of 27f (5′-AGA GTT TGA TCC TGG CTC AG-3′) and 1492r (5′-TAC GGC TAC CTT GTT ACG ACT-3′) [29]. PCR products were purified using a Gene-JET™ PCR Purification Kit (Thermo K0701), and then were sequenced on GATC Company (Ebersberg, Germany) with an ABI 3730xl DNA sequencer using the forward and reverse primers. The bacterial 16S sequences in this study were deposited in GenBank under the accession number MT534272. The obtained sequences and those of their most closely related taxa retrieved from GenBank were aligned using the CLUSTAL X program [30]. Phylogenetic distances were calculated using Kimura’s two-parameter model [31], evolutionary trees were inferred using the neighbor-joining method [32], and the phylogenetic tree was created using the MEGA v6.1 software, with confidence testing by bootstrap analysis (1000 repeats).

### 2.4. Extracellular Biosynthesis of Silver Nanoparticles (Ag-NPs)

Ag-NPs were biologically synthesized by using pure AgNO_3_ (Sigma-Aldrich) as a metal precursor, with the biomass filtrate of isolated actinobacteria as the reducing agent.

Practically, three disks (0.8 mm) of freshly grown cultures of the isolated endophytic actinobacteria were inoculated into 100 mL of starch casein broth (SCB) media and incubated at (30 ± 2) °C for 96 h in an orbital shaker (180 rpm). Then, the actinomycetes biomass was harvested, by passing the SCB media through four layers of compact gauze. The biomass obtained was washed with sterile distilled water, in order to remove leftovers from the growth media. The washed biomass was suspended in 100 mL of distilled water at (30 ± 2) °C for 72 h. After incubation, the mixture was filtered through filter paper (Whatman No. 1) in order to obtain the biomass filtrate, which was used as the reducing agent for the biogenic synthesis of Ag-NPs as follows: 

First, 1% (*v/v*) of 1 mM AgNO_3_ was added to 100 mL of the biomass filtrate and incubated for 24 h., at (30 ± 2) °C in dark and static conditions. The pH of the mixture was adjusted to 9 by 1 N NaOH, which was added in a drop-wise manner under stirring conditions. Actinomycetes biomass filtrate and AgNO_3_ were also used as controls. The synthesis of Ag-NPs was preliminarily examined by a color change from colorless into dark or yellowish-brown and optical density measurements in the range (300–700) nm (JENWAY 6305 Spectrophotometer,230 V/50 Hz, Staffordshire, UK) [33]. The surface Plasmon resonance (SPR) for biogenic Ag-NPs was detected after intervals (1, 3, 7, 14, and 30) days, in order to check the stability of NPs. The biomass filtrate was used as blank for the JENWAY 6305 Spectrophotometer.

### 2.5. Characterization of Biosynthesized Ag-NPs

The shapes and sizes of the biogenic Ag-NPs were identified by transmission electron microscopy (TEM—JEOL 1010, Tokyo, Japan). Briefly, a drop of NPs suspension solution was put on a carbon-coated copper TEM grid. Extra NP solution on the TEM grid was eliminated by touching it to blotting paper. The grid was then kept at room temperature for drying [34]. The binding properties of silver nanoparticles were investigated by FT-IR analysis (Agilent system Cary 630 FTIR model, USA) in the range 400–4000 cm^−1^. The X-ray diffraction (XRD) patterns of silver nanoparticles were analyzed using Shimadzu Scientific Instruments (SSI), Kyoto, Japan. The X-Ray diffraction types of Ag-NPs were obtained with the XRD–6000 series, including residual austenite quantitation, stress analysis, crystallite size/lattice strain, crystallinity calculation, and materials analysis by overlaid XRD (Shimadzu apparatus) with nickel filter and Cu–Ka target. The 2θ values were measured in a range from 4° to 90°. The NPs sizes obtained from XRD analysis were estimated by Scherrer’s formula:

D = 0.94λ/β cos *θ*

where D is the mean particle size, 0.94 is the Scherrer’s constant, λ is the X-ray wavelength, β is the half of the maximum intensity, and θ is the Bragg’s angle.

Furthermore, investigation of the distribution, size, and poly-dispersity index (PDI) of biogenic Ag-NPs in the colloidal solution was carried out using a dynamic light scattering (DLS) technique. The biogenic Ag-NPs were suspended in distilled H_2_O and exposed to a Zeta sizer nano-series (Nano ZS, Malvern, UK). Furthermore, the stability of actinobacterial mediated Ag-NPs synthesis was analyzed using Zeta Potential measurement.

### 2.6. Antibacterial Activity of Ag-NPs 

The antibacterial activity of the biogenic Ag-NPs was examined against five selected bacterial pathogens: two Gram-positive strains (*Bacillus subtilis* ATCC 6633 and *Staphylococcus aureus* ATCC 6538) and three Gram-negative strains (*Salmonella typhimurium* ATCC 14028, *Pseudomonas aeruginosa* ATCC 9022 and *Escherichia coli* ATCC 8739). Freshly grown cultures of the selected bacterial strains were individually prepared in sterile saline solutions at a concentration of (1.5 × 10^8^) CFU/mL, seeded in Mueller-Hinton agar media. According to the agar well diffusion method, 3 wells (0.8 cm diameter) were cut in the seeded Mueller-Hinton plates and 100 µL of Ag-NPs (100 ppm) was added into each well. The plates were kept in the refrigerator for 1 h, then incubated for 24 h at 37 °C. The diameter of the potential growth inhibition zones around each well was recorded (mm). Different Ag-NP concentrations (6.25, 12.5, 25.0, 50.0, and 75.0 ppm) were prepared, in order to determine the minimum inhibitory concentration (MIC) against each bacterial pathogen [35]. The experiments were carried out in triplicates.

### 2.7. Cytotoxic Activity of Ag-NPs Against Cancer and Normal Cells

The potential cytotoxicity of the actinobacterial-synthesized Ag-NPs was assessed by the MTT [3-(4, 5-dimethylthiazol-2-yl)-2, 5-diphenyl tetrazolium bromide] assay using human colorectal adenocarcinoma cells (Caco-2) and normal Vero cells (kidney of African green monkey) procured from ATCC. These cancerous and normal cell lines were chosen as a model for cancerous and normal cell lines, respectively. The cells were cultured in 96-well plates at a concentration of (1 × 10^5^) cells/well and treated with double-fold dilutions of the biologically synthesized Ag-NPs (3.9–1000 μg/mL). After 48 h of incubation, morphological changes that occurred in epithelial cells as a result of treatment with silver nanoparticles were monitored using an inverted light microscope (Nikon, Tokyo, Japan). For the MTT assay, cancer and normal cells were grown in 96-well plates at (1 × 10^5^) cells/well and treated with a series of double-fold concentrations (3.9–1000 μg/mL) of the biogenic Ag-NPs and incubated at 37 °C for 48 h. MTT (5 mg/mL in phosphate-buffered saline) was added to each well and incubated at 37 °C for 1–5 h at 37 °C with 5% carbon dioxide. A purple formazan crystal was formed, which was dissolved by adding dimethyl sulfoxide (10%). Plates were kept in a plate shaker for 30 min in the dark. Finally, the optical density of the samples was measured using a multi-well ELISA plate reader at 560 nm [36]. The percentage of cell viability was calculated using the following formula:

Cell viability % = (sample absorbance/control absorbance) × 100.

### 2.8. Application of Ag-NPs for Medical Fabrics

#### 2.8.1. Loading the Biogenic Ag-NPs onto the Cotton Textile Using the Pad–Dry–Cure Method

Cotton fabrics were washed with warm water and dried. The cotton textile was then cut into pieces (15 cm × 30 cm) and soaked in an aqueous solution of biogenic Ag-NPs (at a safe concentration based on the cytotoxicity tests). For the subsequent processing of fabrics with colloidal silver, the solution was constantly stirred. Fabric samples were immersed in the colloidal bath for 1 min and then thoroughly squeezed under constant pressure using a laboratory pad. Then, the samples were dried at 70 °C for 3 min, and ironed at 150 °C for 2 min. The experiment was conducted using the following treatments: (1) non-processed fabrics as a control, (2) fabrics treated with Ag-NPs, and (3) fabrics treated with Ag-NPs and washed five or ten repeated wash cycles. Each washing cycle lasted 45 min in a warm-water washing machine containing 2% sodium carbonate. At the end of the washing cycle, the fabric was dried in a dryer at 80 °C. 

#### 2.8.2. Scanning Electron Microscopy for Nanocoated Fabrics

The surface characteristics of the treated fabrics and their metal nanoparticle contents were evaluated using SEM (JSM-5400, JEOL, Tokyo, Japan) coupled with energy-dispersive X-ray spectroscopy (EDX).

#### 2.8.3. Antibacterial Activity of Nano-Coated Fabrics

The antibacterial potential of nano-coated fabrics was assessed against bacterial pathogens: Gram-positive strains (*Bacillus subtilis* ATCC 6633 and *Staphylococcus aureus* ATCC 6538) and Gram-negative strains (*Pseudomonas aeruginosa* ATCC 9022 and *Escherichia coli* ATCC 8739). Each bacterial pathogen was seeded into Mueller-Hinton agar media and left to solidify. Then, 1 cm squares of each fabric sample were aseptically prepared, and put onto the agar surface, and incubated for one day. After incubation, the plates were examined, and the diameters of the growth inhibition zones (mm) were recorded around the fabric samples. The experiment was carried out using the following treatments: (1) untreated fabrics as a control, (2) fabrics treated with Ag-NPs, and (3) fabrics treated with Ag-NP and washed 5 and 10 repeated wash cycles.

### 2.9. Statistical Analysis

All results presented in this study are the means of three independent replicates. Data were subjected to analysis of variance (ANOVA) by the statistical package SPSS v17. The mean difference comparison between the treatments was analyzed by the Tukey HSD test at a significance level of *p* ≤ 0.05.

## 3. Results and Discussion 

### 3.1. Isolation and Molecular Identification of Endophytic Actinomycetes

**Figure 1:**
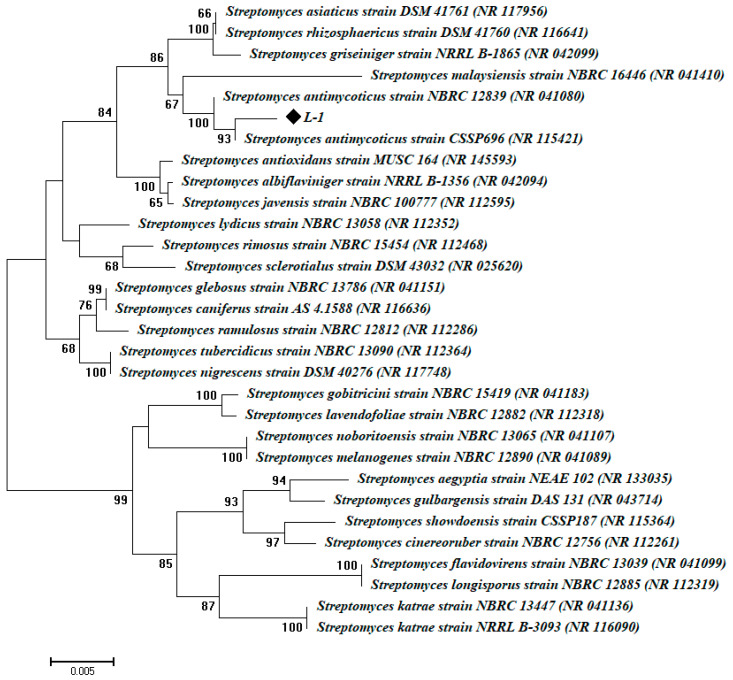
Phylogenetic tree of *Streptomyces antimycoticus* based on 16S rRNA sequences analysis. The symbol◆ indicates to 16S rRNA fragments of the endophytic actinomycetes strain obtained in the current study. The analysis was completed with MEGA 6 using the neighbor-joining method.

Medicinal plants are considered as adequate shelters for several endophytic microbes, including bacteria, fungi, and actinomycetes [14]. Therefore, the isolation of these endophytic microbes, especially from plants inhabiting harsh environments, opens up promising strategies for different biotechnological applications. In this study, an endophytic actinobacterial isolate of L-1 was obtained from healthy leaves of *Mentha longifolia* L.; based on BLAST analysis, the endophytic actinomycetes isolate obtained in this study was identified as *Streptomyces antimycoticus* L-1, with similarity percentage of 98% related to *Streptomyces antimycoticus* (accession number: NR_041080). The phylogenetic tree of 16S rRNA sequences showed homology of the obtained sequence to *Streptomyces antimycoticus* with a bootstrap percentage of more than 90% (Figure 1). To the best of our knowledge, this is the first report of the isolation of *Streptomyces antimycoticus* from the medicinal plant *M. longifolia* for use as a biocatalyst for Ag-NPs synthesis.

### 3.2. Biogenic Synthesis of Ag-NPs

The biogenic fabrication of the nanoscale particles is preferable to physical and chemical processes, due to its properties of energy efficiency, ease of scaling-up, low coast, low toxicity, and biocompatibility. Therefore, biogenic nanoparticles can be safely exploited in many biological applications [37]. The actinomycetes, particularly *Streptomyces*, are some of the best candidates for the large-scale biological fabrication of nano-sized materials, as a result of its cost-effectiveness due to its rapid growth, high biomass production, and environmental friendliness [38]. Furthermore, actinobacterial endophytes extracellularly produce active metabolites, such as enzymes and proteins, which may participate in the synthesis of nanoparticles [20].

**Figure 2:**
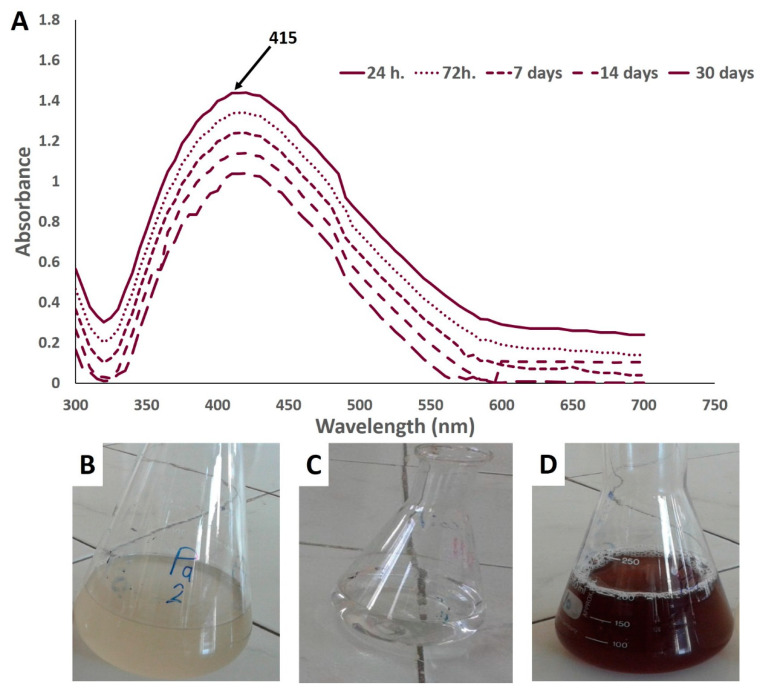
UV–vis spectroscopic analysis and color change of silver nanoparticles (Ag-NPs) synthesized by endophytic *S. antimycoticus* L-1: (**A**) UV–Vis spectra of Ag-NPs at different interval times; (**B**) biomass filtrate color; (**C**) silver nitrate solution; (**D**) yellowish-brown color of Ag-NPs synthesized by *S. antimycoticus* L-1.

In this work, an aqueous solution of AgNO_3_ (1 mM) was added to the washed actinomycetal biomass filtrate. The reaction mixture turned into a yellowish-brown suspension (Figure 2B–D) due to the surface plasmon resonance, confirming the reduction of Ag^+^ ions into nanoscale silver particles. The endophytic actinomycetes biomass filtrate and AgNO_3_ solution did not show any color change after the same incubation period. The reduction efficacy could be attributed to the liberated electron from reducing NO_3_ to NO_2_ and then Ag^+^ to Ag^0^; therefore, the color intensity (in most cases) was related to the number of reduced Ag ions [34].

### 3.3. Characterization of Biosynthesized Silver Nanoparticles

#### 3.3.1. UV–Vis Spectroscopy 

According to UV–vis spectroscopy of the mixture after 24 h of incubation, the maximum observed peak in this study was at 415 nm, which is closely related to spherically shaped NPs [39]. The optimum peaks for green-synthesized Ag-NPs are in the range of 400–460 nm, which is the corresponding absorption range of SPR for Ag-NPs (Figure 2A) [40,41]. The brownish or yellowish-brown color is formed as a result of excitation of surface plasmon resonance, which is particular for Ag-NPs as recommended previously [9,21,42]. The previous studies reported that the surface plasmon resonance showed at 420 nm is usually related to nano-size from 2 nm to 100 nm [21,43]. To check the stability of the NPs solution, the SPR for Ag-NPs synthesized by endophytic *Streptomyces antimycoticus* L-1 did not exhibit any change over different interval times, which confirmed the long-term stability of the biosynthesized Ag-NPs (Figure 2A).

#### 3.3.2. FT-IR Analysis

**Figure 3:**
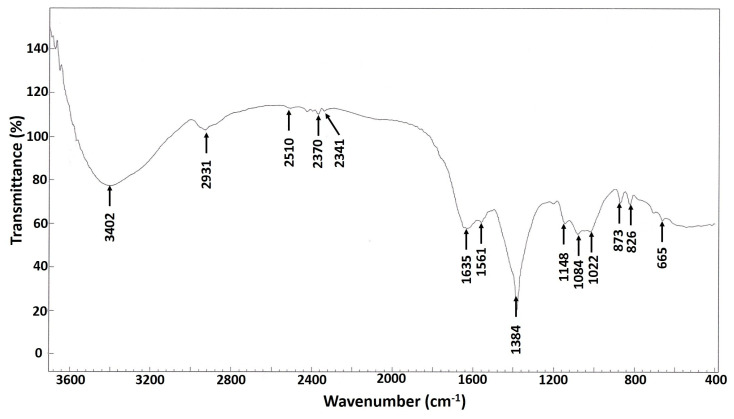
FT-IR spectrum of the Ag-NPs derived from endophytic *S. antimycoticus* L-1.

The FT-IR analysis gives orientation information related to the structure of molecules. Figure 3 showed the FT-IR spectra of biosynthesized Ag-NPs at wavenumber range from 4000–400 cm^−1^. However, many functional groups that played roles in the reduction and capping of the biogenic Ag-NPs were present. The broad band at 3402.0 cm^−1^ is corresponded to O–H and NH stretching vibrations, which were overlapped [44,45]; while the peak at 2931.0 cm^−1^ was assigned to CH aliphatic stretching of hydrocarbon [46,47]. The two peaks at 1635.0 cm^−1^ and 1561.0 cm^−1^ were related to C=O stretching overlapped with amine-bending vibration [46,47]. The peaks at 2370.0 cm^−1^ and 2341.0 cm^−1^ were related to carbon dioxide peaks, adsorbed onto the protein surface [48]. The peak at 1384.0 cm^−1^ was assigned to C–N and –OH bending vibrations [49], which was also emphasized by the two peaks at 1148.0 cm^−1^ and 1084.0 cm^−1^ which are associated with C–C, C–N stretching, as well as symmetric C–O stretching [50]. The peaks at 873.0 cm^−1^ and 665.0 cm^−1^ were related to (CH_2_)n-rocking, associated with out-of-plane OH bending [49,51].

The peaks in the IR spectrum could be attributed to the presence of functional groups (e.g., C–N, –OH, C=O, C–C), which may have formed due to the interactions of metabolites (e.g., proteins) with the biofabricated Ag-NPs. These results are consistent with those obtained by El-Naggar et al. [52], and Sastry et al. [53], who reported that the functional groups (or peaks) such as C=O and C–C arise from heterocyclic compounds, such as proteins, which act as capping agents for NPs.

#### 3.3.3. TEM Analysis

**Figure 4:**
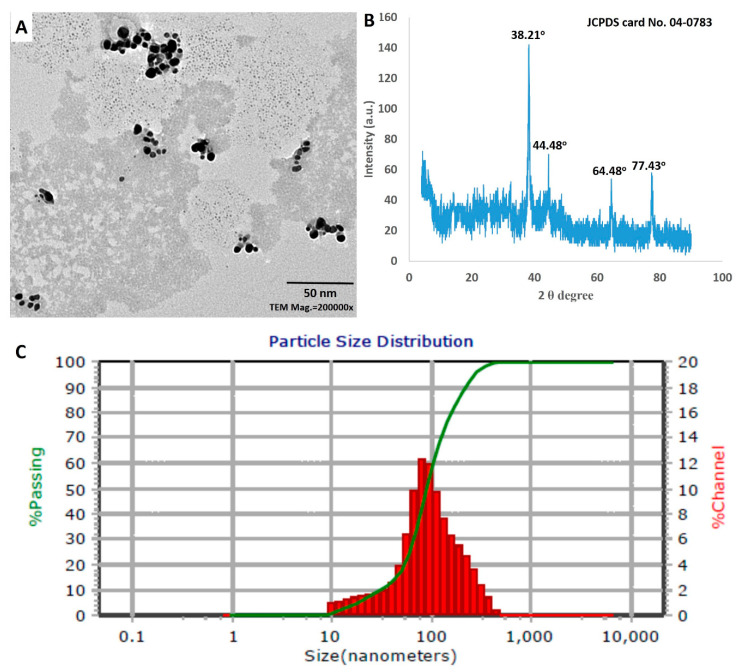
Characterization of Ag-NPs synthesized by *Streptomyces antimycoticus* L-1: (**A**) transmission electron microscopy (TEM) image; (**B**) X-ray diffraction (XRD) analysis; (**C**) dynamic light scattering (DLS) analysis.

The TEM images illustrate the structural qualities of the *S. antimycoticus* derived Ag-NPs (Figure 4A). The biogenic nano-scaled particles exhibit a spherical shape with size variation between 13 nm and 40 nm (Figure 4A), where the nanoparticles are monodispersed without any aggregation. The biofabricated nanoparticles were identical in shape and size to those in previous studies [54,55]. In agreement with our results, Hamouda et al. [56], demonstrated the synthesis of spherical monodispersed silver nanoparticles from a cyanobacterial aqueous extract, which had a dual role in both stabilizing and reducing silver nanoparticles.

#### 3.3.4. XRD Analysis

The structural information of the biogenic Ag-NPs was confirmed by the XRD analysis. The XRD pattern showed the main reflection planes at the 2θ degrees of 38.21°, 44.48°, 64.58°, and 77.43°, corresponding to the lattice plane clusters of (111), (200), (220), and (311), respectively (Figure 4B). The XRD spectrum was compared with standard powder diffraction (JCPDS card no. 04-0783), in order to ensure that the particles in the sample were silver nanoparticles with the characteristic face-centered cubic (FCC) structure of silver crystals [57]. In this study, the plane (111) is the dominant between Ag spheres. This result was compatible with Railean-Plugaru et al., who reported that peak 111 was the dominant among Ag spheres synthesized by actinobacterial *Streptacidiphilus* sp. strain CGG11n [22]. The mean crystalline size of biogenic Ag-NPs was estimated by the Debye–Scherrer’s equation. 

Analysis of the data showed that the crystalline size of the biologically synthesized Ag-NPs was in the range of 19–35 nm, with an average size ~27 nm. The diffractogram contained weak diffraction peaks, which could be attributed to the bio-organic molecules on the surface of the silver nanoparticles [58]. In agreement with our results, Abd-Elnaby et al. [59] reported the extracellular synthesis of silver nanoparticles from marine *Streptomyces rochei* MHM13 with the particle size range of 22–85 nm.

#### 3.3.5. DLS Analysis

The size of Ag-NPs in the colloidal solution was estimated by dynamic light scattering analysis, through the reaction of light beams with the biosynthesized NPs [60]. The diameter of nanoparticles in the colloidal solution (100%) was approximately 90.2 nm (Figure 4C). The size of Ag-NPs acquired from DLS analysis was bigger than those attained from TEM and XRD analyses. These results could be attributed to the size assembled from DLS depending on actinobacterial metabolites that had accumulated on the particle surface, which were responsible for reducing, capping, and stabilizing. Furthermore, the metallic core and non-homogeneous particle distributions have been correlated to bigger NPs sizes under DLS [60,61]. On the other hand, through the assessment of PDI values, DLS analysis provides an articulation about the homogeneity of NPs in colloidal solutions. The homogeneity of the NPs solution is increased or decreased if PDI value is more or less than 0.4, while it is heterogeneous if the PDI value is ≤1. The obtained data highlighted that the PDI value of the biosynthesized Ag-NPs was equal to 0.5. Furthermore, the stability of the biogenic Ag-NPs was distinguished by zeta potential analysis (ζ) through the measurement of surface particle charge. In this study, the ζ value for Ag-NPs was recorded as −19.2 mV, which reflects the stability of the biosynthesized NPs solution. These results are compatible with those of other UV–vis spectroscopy analyses, in which the ζ values of Ag-NPs synthesized by *Streptomyces noursei* H1-1 and *Trichderma longibrachiatum* were −18.9 mV, and −19.7 mV, respectively [9,62].

### 3.4. Antibacterial Activity of Ag-NPs

**Figure 5:**
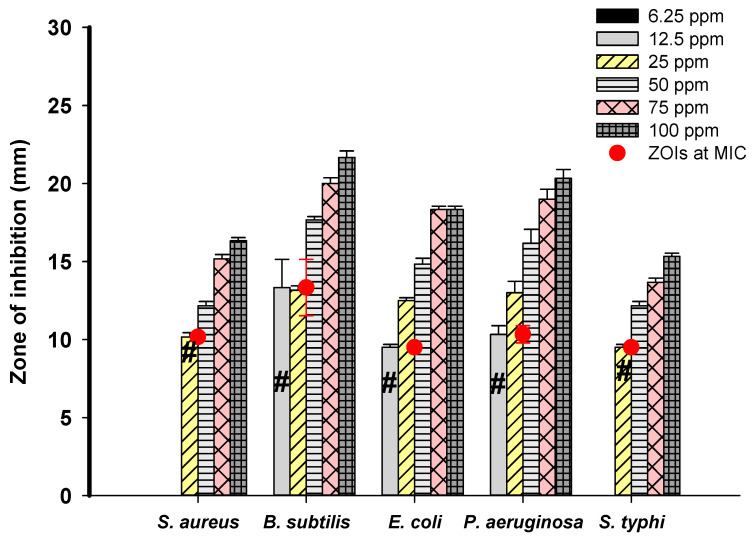
Antibacterial activities and minimum inhibitory concentrations (MICs) for Ag-NPs synthesized by *S. antimycoticus* L-1 against pathogenic *S. aureus, B. subtilis, P. aeruginosa, E. coli,* and *S. typhimurium.* # indicates MIC value.

The antibacterial performance of the biogenic silver nanoparticle suspension was evaluated against selected pathogenic bacteria using the well-diffusion assay. The investigated bio-colloidal silver offered auspicious nano-antibiotic properties, with broad-spectrum activity against both Gram-negative and Gram-positive pathogenic bacteria. Evaluation of the antibacterial activity of Ag-NPs by an agar well diffusion test has been previously carried out [63]. Silver nanoparticles at 12.5 ppm exhibited inhibitory effects against *Bacillus subtilis, Escherichia coli* and *Pseudomonas aeruginosa*, forming inhibition zones (ZOI) of (13.3 ± 0.6), (9.5 ± 0.4), and (10.3 ± 0.3) mm, respectively. It has been proven that the antibacterial activity of silver nanoparticles depends on the concentration. For example, at a Ag-NP concentration of 25 ppm, higher activities were seen against bacterial pathogens, with recorded ZOIs of (13.6 ± 0.7), (12.5 ± 0.4), and (13 ± 0.8) mm corresponding to *Bacillus subtilis, Escherichia coli*, and *Pseudomonas aeruginosa*, respectively. Moreover, *Staphylococcus aureus* and *Salmonella typhimurium* showed sensitivity to bio-colloidal silver at 25 ppm, with recorded ZOIs of (10.16 ± 0.7) and (9.5 ± 0.4) mm, respectively. The *Streptomyces*-derived Ag-NPs showed enhanced antibacterial activity against all tested pathogens at 50 ppm, and 75 ppm. The maximum antibacterial activity was seen with the highest nano-silver concentration of 100 ppm, which exhibited the highest ZOIs values of (16.33 ± 0.5), (21.7 ± 1.0), (18.3 ± 0.5), (20.3 ± 1.4) and (15.3 ± 0.6) mm for *Staphylococcus aureus, Bacillus subtilis, Escherichia coli, Pseudomonas aeruginosa*, and *Salmonella typhimurium*, respectively. Moreover, *Bacillus subtilis* presented the highest sensitivity to the silver nano-antibiotics, while the lowest sensitivity was assigned to *Salmonella typhimurium*. Bio-colloidal silver synthesized from actinomycetes have exhibited an efficient nano-antibiotics and antibacterial activities [64]. Corresponding with our investigations, the well diffusion method was used to evaluate the antibacterial properties of *Streptomyces* sp-induced silver nanoparticles, which displayed good antibacterial activity against Gram-negative and Gram-positive bacteria, including methicillin-resistant *Staphylococcus aureus* (MRSA) [65]. The antibacterial potential of the silver nanomaterials from *Streptomyces parvus* against various pathogens, including *S. aureus, E. coli, E. faecalis, P. aeruginosa* and *K. pneumoniae*, has been recently reported [66]. The antibacterial properties of biogenic Ag-NPs may be attributed to different mechanisms, such as charge influence of Ag-NPs. The partial dissolution of the bacterial cell membrane may be achieved due to the electrostatic attraction between the negative charge of the cell wall and the positive charge of the silver nanomaterial or by the interaction of nano-scaled silver with phosphorylated or sulfur proteins of the bacterial cell wall, causing the destabilization and depolarization of bacteria membrane to lose its integrity and leakage of H^+^ [67]. Anther mechanism of biological toxicity of Ag-NPs could be attributed to the release of Ag^+^ through oxidation process. In this mechanism, Ag-NPs act as a carrier for Ag^+^, once liberated, Ag^+^ reacts with phosphate, thiol group of cysteine amino acids, glutamine amino acid, and carboxyl group, leading to bacterial death [68,69]. Moreover, the biocidal effect of Ag-NPs could be attributed to elevated ROS as a result of Ag^+^ release, which destroys cellular respiration enzymes and the ultimate result is cell death [46]. Recently, Alsharif et al. [9] reported the extracellular synthesis of Ag-NPs by *Streptomyces noursei* H1-1 and declared the potential antibacterial activity of these nanoparticles in low concentrations (25 ppm and 50 ppm) against the selected pathogens *S. typhimurium, P. aeruginosa, E. coli, B. subtilis*, and *S. aureus*. In the same context, Wypij et al. [70] reported the biogenic synthesis of Ag-NPs from *Streptomyces xinghaiensis* OF1 with efficient nano-antibiotic properties against *B. subtilis, S. aureus, E. coli*, and *P. aeruginosa*. Dong et al. [25] announced the bacteriostatic action of biosynthetic Ag-NPs against *Vibrio Natriegens*; additionally, they indicated an inverse relationship between the size of nanoparticles and their antibacterial activity. Our results revealed that the *Streptomyces*-derived Ag-NPs were active at low concentration (25 ppm) against all selected pathogens. The MIC values of biosynthesized Ag-NPs against *S. aureus, B. subtilis, E. coli, P. aeruginosa,* and *S. typhimurium* were 25.0 ppm, 12.5 ppm, 12.5 ppm, 12.5 ppm and 25.0 ppm, respectively, with ZOIs of (10.16 ± 0.7), (13.3 ± 0.6), (10.3 ± 0.3), (9.5 ± 0.4) and (9.5 ± 0.4) mm as shown in Figure 5. Hence, Ag-NPs have been shown to manifest powerful antibacterial activities, which might be clinically useful for controlling various bacterial infections. 

### 3.5. Cytotoxic Activity of Ag-NPs Against Cancer and Normal Cells

**Figure 6:**
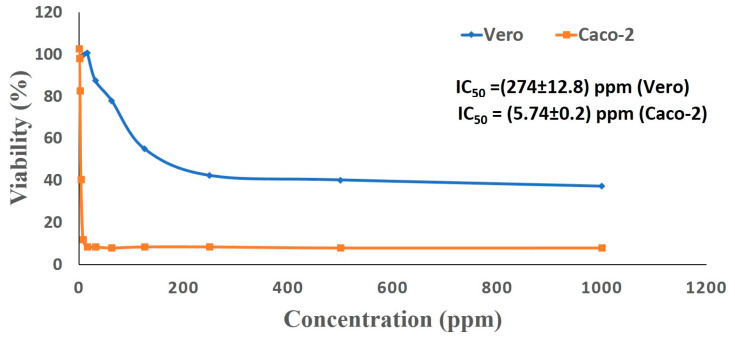
Cytotoxic activity of Ag-NPs derived from *S. antimycoticus* L-1 against Vero and Caco-2 cell lines.

Silver nanoparticles play a paramount role in nano-medicine, with potential for use as antibacterial or anticancer agents [71,72]. Interestingly, biologically derived nanoparticles are more effective in biomedical applications, as compared to those manufactured by physical or chemical procedures [73,74]. Diverse anticancer drugs have been developed using nanotechnology; for example, Myocet™ (Perrigo, Dublin, Ireland), Abraxane^®^ (Celgene, Summit, NJ, USA) and Doxil^®^ (Johnson and Johnson, New Brunswick, NJ, USA) have been recently approved by the Food and Drug Administration (FDA) for clinical applications [75]. Accordingly, the current study was designed to assess the potential anticancer activity of the biosynthesized Ag-NPs. Therefore, an MTT experiment was performed to estimate the cytotoxic effect of Ag-NPs synthesized by *S. antimycoticus* against the cancerous Caco-2 (human colon cancer) and normal Vero (kidney of African green monkey) cell lines. MTT is an accurate colorimetric method that is commonly used to evaluate cellular toxicity, as well as cell proliferation and viability. The microscopic investigation of cell lines exposed to Ag-NPs exhibited the cytotoxicity of nanoparticles against mammalian cells, which resulted in the loss of the characteristic monolayer of these epithelial cells. Moreover, we found that the cytotoxic properties of Ag-NPs were dose-dependent. The reduced viability of cancerous caco-2 cell lines could be attributed to the apoptotic effect induced by the biogenic silver nanoparticles. However, other mechanisms for cytotoxicity of Ag-NPs have been studied, such as modifying the mitochondrial membrane potential, DNA fragmentation, leakage of lactate dehydrogenase (LDH), activation of apoptotic caspases, ROS generation and nuclear fragmentation [76]. In agreement with our results, the *Streptomyces noursei* H1-1-based Ag-NPs manifested a functional dose-dependent cytotoxic effect against cancerous cell lines, where very low concentrations (5.6 ± 3.0 μg/mL) of Ag-NPs inhibited 50% of caco-2 (cancer cell line); while the normal Vero cell line, when exposed to (511.7 ± 68.5) μg/mL of bio-silver nanoparticles, halved their cell viable cell count [9]. Recently, Liao et al. [77] have reported the dose, time, and size dependency of the Ag-NPs mediated cytotoxicity, mainly for particles smaller than 10 nm. According to our results, the nano-processed cell lines lost their adherent ability, as evidenced by the complete or partial breakdown of the monolayer integrity, as well as shrinking, floating, and cells becoming granular and round. Similar morphological modifications were obtained after incubating human cancer cells (Caco-2) and normal cell lines (WI 38) with biogenic Selenium nanomaterials, which led to modified cell shape, rounding, granulation, shrinking, and the loss of monolayer integrity in cloned cell [78]. The cytotoxic abilities (IC_50_) of the biogenic Ag-NPs (values for 50% inhibition of cell viability) were calculated graphically (Figure 6). The toxicity of biogenic Ag-NPs against human colon carcinoma cell line (HCT-116) and breast cancer cell line (MCF-7) has been determined, recording the IC_50_ values of 5.4 µg/mL and 6.1 µg/mL, respectively [56]. In the current study, the normal Vero cells recorded a higher IC_50_ value (274.8 ± 12.8 ppm) than the value for the cancerous Caco-2 cell line (5.74 ± 0.2 ppm). The lowered effect of the *Streptomyces-*derived Ag-NPs on the Vero cells, compared to its potent impact on the Caco-2 tumor cell line, revealed its selective activity toward the carcinoma cell line and, thus, its potential for clinical use as a safe therapeutic agent. According to data of the antibacterial activity and in-vitro cytotoxicity, we can select a safe dose to apply on cotton fabrics. 

### 3.6. Application of Ag-NPs for Medical Fabrics

#### 3.6.1. Loading Biogenic Ag-NPs onto Cotton Textile

**Figure 7:**
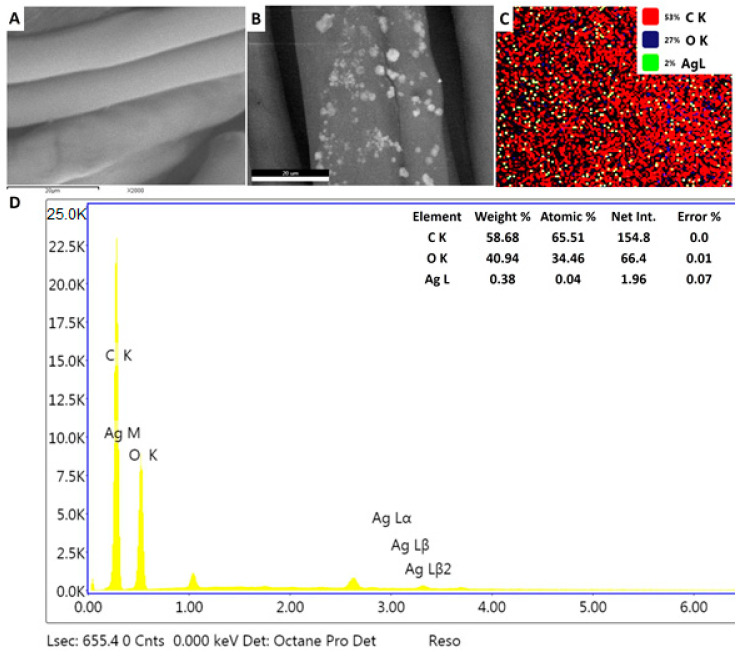
Scanning electron microscopy results for cotton fabrics; (**A**) cotton fabric without any treatment, and showing smooth surface; (**B**) cotton fabrics treated with Ag-NPs (100 ppm), showing the deposited NPs on the cotton surface; (**C**) mapping picture of treated fabrics with Ag-NPs, showing qualitative elemental analysis; (**D**) EDX of the treated sample with elemental analysis of the Ag-NPs contents.

Cotton fabric pieces were washed with warm water and subjected to a simple pad technique, in order to be loaded with a solution of the biologically manufactured Ag-NPs (100 ppm); this concentration was deemed safe for the normal cell line (based on our investigations of cytotoxicity). The color of treated fabrics by a safe Ag-NPs dose tends to be a pale yellow. Washing and flooding fabrics with water before nano-finishing composes a negative charge on the surface of the textile, such that the positively charged Ag-NPs would be electrostatically attracted to that negative charge on the fabric surface [79]. The surface morphology of textile samples was then examined by SEM. The blank fabric showed a refined overlapping fibrous frame with a soft surface, without any contaminants or depositions (Figure 7A). As for the treated fabric, although it seemed to retain its structure, its surface was covered with Ag-NPs that were incorporated into the tissue fibers and evenly dispersed across the surface of the textile (Figure 7B,C). In this respect, Xing et al. [80] reported the effective deposition of nano-sized silver, which was homogeneously distributed on the textile surface. In the present study, our characterization of the *Streptomyces*-derived Ag-NPs revealed their small size (13–40 nm), which could support their adhesion to the fabric surface, as it is expected that smaller particles can penetrate deeply into the fabric and tightly adhere to the textile fibers. Furthermore, EDX spectroscopy determined the chemical elements of the nano-finished fabrics, declaring peaks corresponding to C and O, as well as an incoming peak monitored around 3 keV, which could be attributed to the nano-scale silver particles. Silver nanoparticles represented about 2% of the total elements on the modified fabrics surface and approximately 0.38**%** of the weight of the surface elements (Figure 7D). Accordingly, the SEM/EDX results confirmed the existence of Ag-NPs attached to the textile surface. In line with the current study, Othman et al. [81] reported the efficient application of bio-fabricated Ag-NPs for functionalizing nano-finished fabrics with antimicrobial properties; their EDX spectra showed a strong peak around 3.0 keV, thus demonstrating the deposition of nano-sized silver on the textile surface. Our results showed that the bio-silver nanoparticles effectively integrated into the surface of the treated fabrics and formed a reinforced surface layer; thus, they can be used as finishing materials for the multifunctional fabrics.

#### 3.6.2. Antibacterial Activity of Nano-Coated Fabrics

Fabrics in contact with the human body provide a moist and warm climate for microorganisms; as such, sweaty clothes are considered a perfect environment for bacterial outgrowth and propagation [82,83]. Therefore, the development of Ag-based antimicrobial fabrics for the treatment of clinical bandages and clothing is a promising solution for biomedical applications. Moreover, nano-finished textiles possess water- and/or oil-repellent properties [84], thus contributing to prevention of microbial growth on the processed fabrics.

The antibacterial activities of nano-finished fabrics have been qualitatively evaluated against selected pathogenic bacteria by investigating zones of inhibition. Our investigations revealed that blank fabric pieces had no antibacterial effects either before or after laundering. On the contrary, the Ag-based biological fabrics exhibited significant antibacterial activity. Although the biogenic colloidal silver was applied at a low concentration, its regular distribution across the fabric surface provided broad-spectrum activity. The silver-coated fabrics presented the highest activity against *B. subtilis,* recording an inhibition zone of (2.7 ± 0.3) mm, while the minimum inhibition zone was registered for *P. aeruginosa* (1.3 ± 0.01 mm). Furthermore, the modified textile displayed significant antibacterial properties versus *E. coli* and *S. aureus,* recording growth inhibition zones of (2.1 ± 0.2), (1.8 ± 0.1) mm, respectively. Washing fastness is a key factor for nano-finished fabrics to remain applicable; it has been noticed that the efficacy of the Ag- fabrics against tested bacteria reduced after washing. However, our results demonstrated the durability of the proposed silver-based fabrics, which retained their biocidal potential after even 10 repetitive washing cycles (Table 1). Nevertheless, Ag-NPs could be abraded from the fabric by the mechanistic vibration and raised temperature during laundry procedures [78]. Therefore, it is expected that the nano-finished fabrics will lose their antibacterial activity after repeated washing. In line with our results, Othman et al. [81] used fungus-derived colloidal silver to functionalize fabrics and reported the activity of the processed fabric against *Candida albicans, Bacillus mycoides,* and *Escherichia coli*, as well as the durability of the nano-finished fabrics and their stability after five washing cycles. Moreover, our data were compatible with those of El-Naggar et al. [52], who reported the efficacy of cotton fabrics loaded with Ag-NPs synthesized by *Streptomyces* sp. strain SSHH-1E against pathogenic Gram-positive, Gram-negative, and yeast species. 

## 4. Conclusions

In this study, healthy leaves of the medicinal plant *Mentha longifolia* L. were collected from Rihibat Nada, Saint Katherine, South Sinai, Egypt, and used for the isolation of endophytic actinobacteria, which was identified as *Streptomyces antimycoticus* L-1 based on 16S rRNA sequencing. The *S. antimycoticus* L-1 strain was used as a biocatalyst for reducing, capping, and stabilizing Ag-NPs. A color change from colorless to yellowish-brown was the first monitored for the green synthesis of Ag-NPs using metabolites involving the biomass filtrate of endophytic *S. antimycoticus* L-1. Physico-chemical Ag-NPs characterizations were achieved by UV–vis spectroscopy, FT-IR, TEM, XRD, DLS and zeta potential analyses. The data confirmed the preparation of crystalline, spherical Ag-NPs with an average size of 13–40 nm at maximum SPR 415 nm, which remained stable for a long time. In addition, our data demonstrated the high stability and homogeneity of Ag-NPs, with a negative surface charge of −19.2 mV and polydispersity index (PDI) equal to 0.5. Analysis of the biological activities of the green-synthesized Ag-NPs revealed their efficacy as bactericidal agents against pathogenic Gram-positive and Gram-negative bacteria, as well as their in-vitro cytotoxicity against a cancerous cell line. Furthermore, cotton fabrics treated with a safe Ag-NPs dose (100 ppm) acquired new functional properties, including self-cleaning against pathogenic bacteria.

## Figures and Tables

**Table 1:** Effect of repeated washing on the antibacterial properties of silver nanoparticle-finished fabrics by qualitative assessment method.

Number of Washing Cycles	Clear Zone (mm)			
	*S. aureus*	*B. subtilis*	*P. aeruginosa*	*E. coli*
**Before**	1.8 ± 0.1 ^a^	2.7 ± 0.3 ^a^	1.3 ± 0.01 ^a^	2.1 ± 0.18 ^a^
**After 5 cycles**	0.9 ± 0.1 ^b^	1.9 ± 0.1 ^b^	0.9 ± 0.03 ^b^	1.8 ± 0.07 ^b^
**After 10 cycles**	0.7 ± 0.1 ^c^	1.5 ± 0.2 ^c^	0.6 ± 0.7 ^c^	0.9 ± 0.05 ^c^

Different letters between columns denote significantly different (*p* ≤ 0.05) mean values, as determined by the Honestly Significant Difference (LSD) test.
